# Association of spring-summer hydrology and meteorology with human West Nile virus infection in West Texas, USA, 2002–2016

**DOI:** 10.1186/s13071-018-2781-0

**Published:** 2018-04-04

**Authors:** Israel Ukawuba, Jeffrey Shaman

**Affiliations:** Mailman School of Public Health, Columbia University, 722 W 168th, New York, NY 10032 USA

**Keywords:** Humidity, Hydrology, Soil moisture, Spring, Summer, Temperature, Texas, West Nile virus

## Abstract

**Background:**

The emergence of West Nile virus (WNV) in the Western Hemisphere has motivated research into the processes contributing to the incidence and persistence of the disease in the region. Meteorology and hydrology are fundamental determinants of vector-borne disease transmission dynamics of a region. The availability of water influences the population dynamics of vector and host, while temperature impacts vector growth rates, feeding habits, and disease transmission potential. Characterization of the temporal pattern of environmental factors influencing WNV risk is crucial to broaden our understanding of local transmission dynamics and to inform efforts of control and surveillance.

**Methods:**

We used hydrologic, meteorological and WNV data from west Texas (2002–2016) to analyze the relationship between environmental conditions and annual human WNV infection. A Bayesian model averaging framework was used to evaluate the association of monthly environmental conditions with WNV infection.

**Results:**

Findings indicate that wet conditions in the spring combined with dry and cool conditions in the summer are associated with increased annual WNV cases. Bayesian multi-model inference reveals monthly means of soil moisture, specific humidity and temperature to be the most important variables among predictors tested. Environmental conditions in March, June, July and August were the leading predictors in the best-fitting models.

**Conclusions:**

The results significantly link soil moisture and temperature in the spring and summer to WNV transmission risk. Wet spring in association with dry and cool summer was the temporal pattern best-describing WNV, regardless of year. Our findings also highlight that soil moisture may be a stronger predictor of annual WNV transmission than rainfall.

## Background

West Nile virus (WNV; family Flaviviridae, genus *Flavivirus*) is a zoonotic pathogen maintained in nature through transmission mainly between *Culex* mosquitoes and various species of birds [1], especially passerine birds [2, 3]. Spillover transmission to humans occurs when infected mosquitoes feed on uninfected human hosts [4, 5], a risk that increases following local virus amplification in both vector and avian host populations. In Texas, the first human WNV case was reported in 2002; since then, WNV has become endemic in the state [6] and cases have been reported annually, with unprecedented outbreaks in 2003 [6] and 2012 [7]. Throughout the temperate world, WNV is seasonal, with the greatest number of cases reported during summer and early fall when temperatures are warmer and the fewest number of cases reported during winter and early spring when temperatures are cooler. Both precipitation and temperature modulate mosquito ecology, and for this reason the effects of temperature and precipitation on WNV transmission dynamics have long been investigated [8–10].

Temperature influences mosquito-host interaction and the potential for disease transmission. Optimal temperatures shorten the number of days between blood meals and egg-laying (i.e. the gonotrophic cycle) [11] in female mosquitoes, increasing their contact rate with hosts (avian and human). Temperature also increases the development rate of the virus in the mosquito (i.e. shorter extrinsic incubation period (EIP)) [12–14], reducing the amount of time it takes for the mosquito to become infectious. Together with decreased gonotrophic cycle (i.e. increased contact rate), a shorter EIP increases the potential for WNV transmission [15]. Moreover, warm temperatures within an optimal range [12] decrease the development time of mosquitoes [16] and lengthens their longevity, further supporting mosquito reproduction and WNV transmission. Indeed, warmer temperatures have been shown to be closely associated with increased transmission [17] and colder temperatures have been linked with declines in mosquito populations [18, 19] and reductions of WNV transmission [17, 20].

Rainfall also impacts WNV transmission. For many mosquito species, rainfall provides the water needed for oviposition and larval habitats [21]. Though rainfall is associated with mosquito habitat availability and consequently WNV transmission, past research indicates that the association between rainfall and WNV disease transmission is not quite straightforward. A lack of rainfall has been shown to be linked to WNV amplification [22, 23]. As wildlife, birds and mosquitoes gather at surviving water pools during droughts, host density increases vector-avian host contact rates, fostering WNV transmission in an otherwise limited setting. Also, during such dry spells, mosquito populations may rise due to decreased predator numbers [24] and standing water on the land surface may become more nutrient-rich and better support *Culex* larvae growth and development. Irrigation offers another route for drought-induced amplification [25, 26], as artificial breeding sites may develop, attracting birds and other wildlife. Humidity, like rainfall, is also important for the mosquito life-cycle. Many *Culex* mosquitoes initiate host-seeking, blood-feeding, and egg-laying in response to increases in near-surface humidity [21, 27].

Several studies have evaluated the association between water-availability and WNV transmission by using rainfall or precipitation. One limitation to this approach is that although rainfall reflects the amount of rainwater received, it lacks feedback from land-related factors acting on water retention. Whereas rainfall only describes water delivered to the land surface, estimates of land surface moisture reflect overall water input and output, which is modulated by other meteorological variables, e.g. temperature, wind speed, solar insolation, as well as topography [28, 29]. To better capture the effects of these additional variables, some studies have used vegetation indices or modeled near-surface soil hydrologic conditions to estimate surface water availability and describe WNV dynamics. Such measures of land surface moisture have been shown to be important predictors of WNV dynamics in some settings [22, 30–32].

Since its emergence in Texas, WNV has been studied with respect to climate and the environment. Efforts to control WNV transmission could benefit from careful monitoring of hydrologic and meteorological conditions [33–35]. For this study, we sought to examine the temporal pattern of meteorology and hydrology that describes the risk of elevated human WNV infection. We used monthly means of temperature, specific humidity, soil moisture and precipitation data resolved at the county scale, to model annual county-level case counts of human WNV. Even though humidity is an additional indicator of water availability, we examine it because it is also a determinant of mosquito life-cycle and disease transmission, as many *Culex* mosquitoes initiate host-seeking, blood-feeding, and egg-laying in response to increases in near-surface humidity [21, 27]. We hypothesized that warmer, wetter spring conditions and drier summer conditions are strongly associated with increased numbers of WNV cases.

## Methods

### Study region

**Fig. 1:**
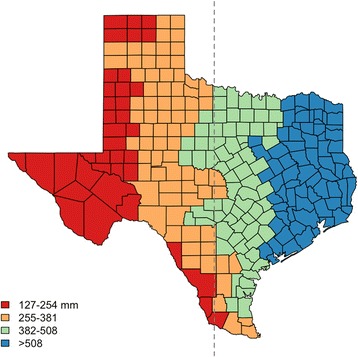
Map of annual precipitation in Texas, 2002 to 2016. Counties left of dashed-line grouped as west Texas (106°14'30"W to 98°30'150"W). Polygons represent counties and their annual precipitation average from 2002 to 2012: red (127–254 mm); orange (255–381 mm); green (382–508 mm); and blue (more than 508 mm)

The region of study is west Texas, delineated as the area of the state between longitudes 106°14'30"W and 98°30'150"W. Mean annual precipitation is between 127 mm in the far west to 380 mm in the central region. Monthly mean precipitation is greatest during May-October. Average monthly temperature can range from near freezing in the winter to 35 °C in the summer. Differences in hydrology between east and west Texas motivate our focus on west Texas (Fig. 1). In the west, Texas is typically arid, mountainous, and filled with grasslands and desert regions, whereas in the east, regions could be lowlands, wet, humid, and forested [36, 37]. Such differences in climate lead to varying natural habitats for birds and other wildlife.

### Human data

**Fig. 2:**
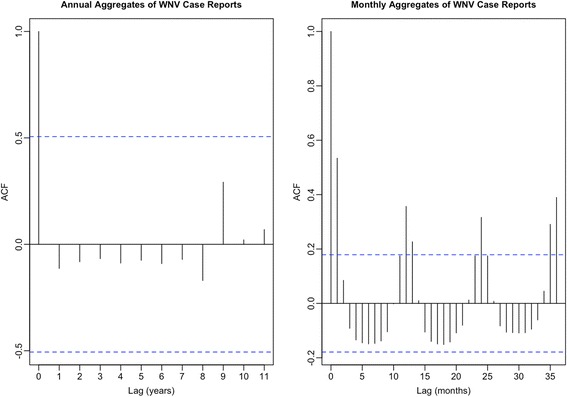
Correlogram of monthly and annual human WNV reports. Dotted blue lines indicate coverage probability of 95% confidence interval (CI), outside which correlations are considered significant. Autocorrelations at 1-month and 12-month lags are significant for monthly cases (95% CI: -0.18–0.18). However, autocorrelation at the annual level is not significant (95% CI: -0.51–0.51). Correlation at the monthly scale may be more meaningful than correlation observed at the annual level

Human WNV cases reported between 2002 and 2016 in west Texas counties were totaled into yearly sums and used as the dependent variable. During the study period, 1269 annual cases of human WNV were reported. By using yearly totals for each county, the temporal autocorrelation apparent in month-month case totals diminishes (Fig. 2). To account for the rate of WNV outcome by population, we used county-level population data for 2000 and 2010 extracted from the US Bureau of the Census website [38].

### Hydrologic/meteorological data

**Fig. 3:**
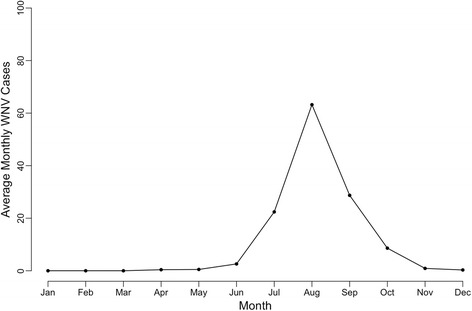
Seasonality of WNV among humans, west Texas, USA, 2002–2016. Transmission onset often begins in June. August typically marks the height of the season

North American Land and Data Assimilation System (NLDAS) records of hourly rainfall (mm/day), specific humidity (kg/kg) and temperature (Kelvin) during 2002–2016 were averaged to monthly means at 1/8th degree resolution (13 × 13 km) [29]. These monthly averages were used as independent variables in a generalized linear model. Although on average, June describes the onset of West Nile virus season in west Texas (Fig. 3), our study focused on hydrologic and meteorological conditions up to three months before the WNV season-from March through August - the peak of the season. We assume a three month-lag relationship between environmental conditions and the onset of West Nile virus transmission [39, 40], and we also assume that conditions between March and August are critical to changes in annual WNV outcome.

Many mosquito species exploit the waters that naturally pool and pond at the land surface as habitats for oviposition and larval development. Actual measurements of land surface wetness are often difficult to acquire but can be simulated using a hydrologic model of the land surface-atmosphere interface [41, 42]. Model-simulated soil moisture can be used as a proxy for breeding site abundance and has been used to predict the abundance of flood and swamp water mosquitoes [28]. NLDAS-2 Mosaic hydrology model estimates of soil moisture levels are generated through dynamical simulation using the NLDAS hourly meteorological data [29]. For land surface water availability, we use the layer 1 soil moisture (L1SM) estimates of the Mosaic model (kg/m^2^), which represents the water content in the top 10 cm of the soil column. To enable geographical comparison with the county-based human WNV data, 13 × 13 km grid of monthly average L1SM and NLDAS meteorological data were interpolated to the centroids of each county in west Texas. Validation of top 10 cm soil column [43] and anomalies of top 40 cm [43, 44] of the Mosaic model show good agreement with observed values in regions of southern Great Plains, next to north-west Texas.

The L1SM estimates represent upper soil column moisture levels in the natural environment. Regions with high urban development may have high percentage of impervious or artificial surfaces. Hence, it may be difficult for estimated hydrology to accurately reflect the development of aquatic habitats in these regions. Impervious  surfaces, such as cisterns, culverts, and storm drains, can house breeding sites, regardless of soil water retaining capacity or actual local surface wetness. Therefore, water availability in some aquatic habitats may be better represented by precipitation variability. Consequently, we include both precipitation and soil moisture in the present analysis.

### Model

**Fig. 4:**
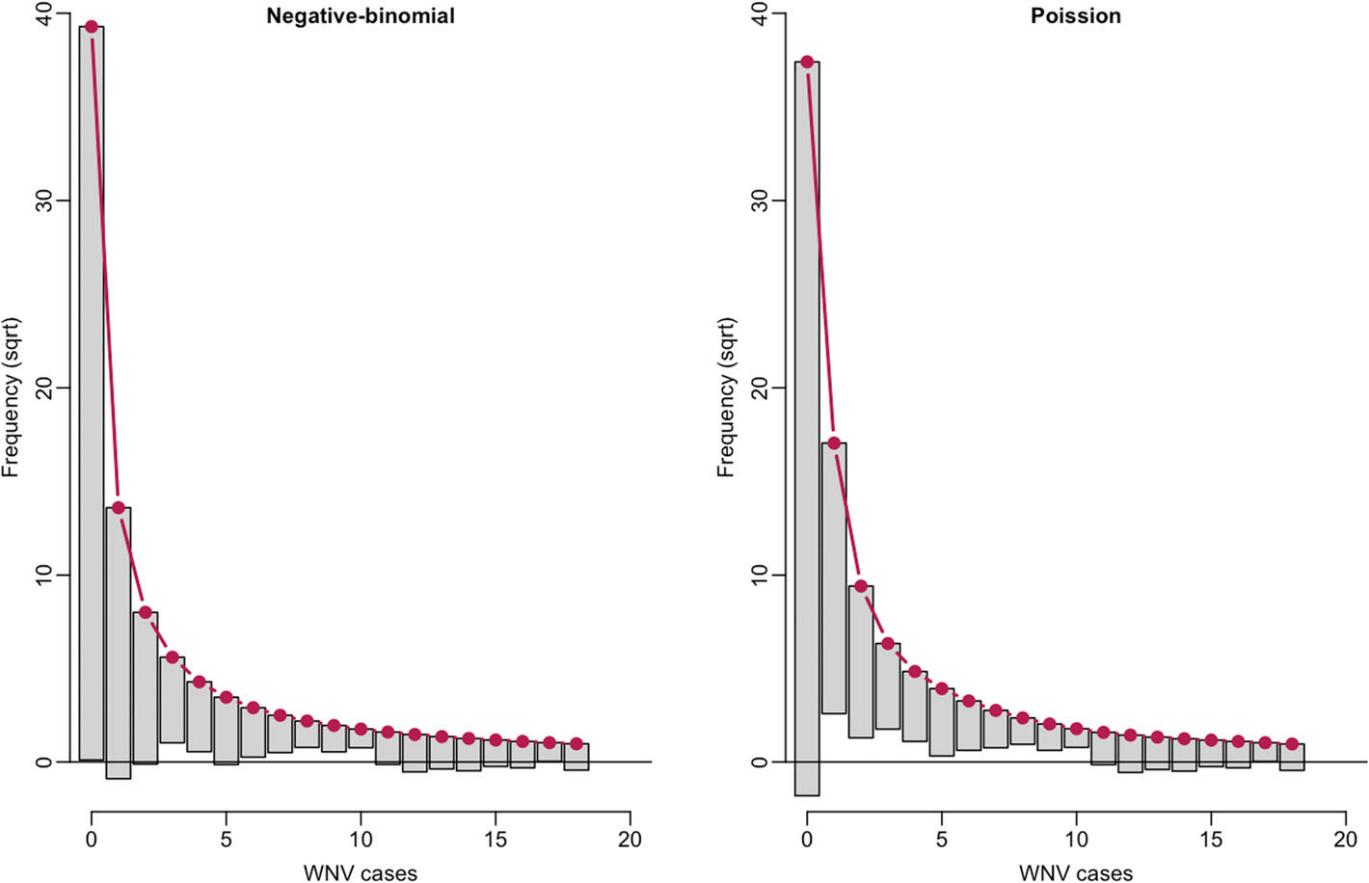
Rootogram [89] comparing the fit of Poisson regression and negative-binomial regression. Left panel negative-binomial, right panel Poisson. The negative-binomial fit shows marked reductions in over-fit and under-fit, especially for counts of zero and counts of 1

**Fig. 5:**
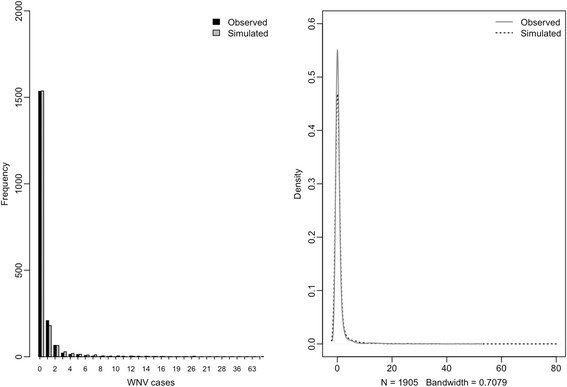
Frequency and density of the negative-binomial distribution of simulated WNV cases. Simulated data are from the average of 300 different simulations, using parameters from the best-fliting model (μ = 8.667714e-92, dispersion parameter = 0.3432, *n* = 1905) compared to observed WNV cases. The difference between simulated data and observed data is small, supporting our choice of negative-binomial model in describing WNV data

To estimate the effects of the independent variables, we used a negative-binomial generalized linear model (GLM) framework. In preliminary analysis, negative-binomial regression showed less over-fitting/under-fitting than Poisson regression (Fig. 4). Data simulated with parameters of top models fitted with negative-binomial framework matched the distribution of the observed data (Fig. 5).

The negative-binomial model form is:

$$ {Y}_{i,t}={\beta}_0+{\mathbf{X}}_{\mathbf{i},\mathbf{t}}\mathbf{B}+{S}_{i,t}+\varepsilon $$

where *Y*_i,t_ is the number of human WNV cases for county *i* during year *t*, *β*_*0*_ is the model intercept, **X**_i,t_ is the matrix of independent variables (e.g. March temperature, April temperature, June L1SM and August rainfall) for county *i* during year *t*, **B** is the vector of parameter estimates, and *ε* is residual error. S_*i,t*_ is an offset term, the natural log of population for county *i* during year *t*. All combinations of March to August rainfall, L1SM, temperature and specific humidity with one to four explanatory variables were tested: a total of *n*!/(*n*-r)! models, where *n* = 24 and *r* = 1, 2, 3 or 4. The null model, which included only the intercept and offset term, was also assessed.

### Statistical analysis

Models were retained for further analysis, provided that all coefficient estimates of variables and the intercept had *P*-values of 0.05 or lower. To rank the retained models (including the null model) based on goodness of fit [45], we used the Bayesian information criteria (BIC). Lower BIC is better.

Use of only the model with the lowest BIC (the top model) may not reflect the general effects of environmental conditions associated with WNV transmission. For example, a model with a slightly higher BIC than the top-ranked model may contain different environmental predictors that fit the data almost as well. For this reason, trusting one single model implies a level of confidence in a limited set of environmental conditions that is less aware of the fitted data [46]. Therefore, we employed multi-model inference or model averaging, an approach which considers possible explanations from competing hypotheses that significantly explain the data. For the averaged model, parameter and standard error estimates are natural averages of variable parameters from the retained competing models set, weighted by the summed weights (wBIC) [47]. During modeling, we addressed the issue of multicollinearity among predictors in the top set of competing models, by using the model averaging function in the *MuMIn* package [48] in R [49] to standardize coefficient estimates by their partial standard deviations [50, 51]. Collinearity is a common issue that occurs when modeling with environmental factors [52, 53], and correlated predictors tend to perplex interpretation of averaged models, due to varying covariance structure in the participating models.

BIC weight (wBIC or *wj*) for a model was computed as follows:

$$ {w}_j=\frac{\exp \left[\hbox{---} \frac{1}{2}{\Delta}_j\right]}{\sum \limits_{j=1}^m\exp \left[\hbox{---} \frac{1}{2}{\Delta}_j\right]} $$

where *∆*_*j*_ = *BIC*_*j*_ − *minBIC*. *BIC*_*j*_ is the BIC value for model *j*, and min*BIC* is the BIC value of the model with the lowest BIC among the set of *m* candidate models. Total wBIC among the *m* candidate models equals 1. The smallest subset of models with a total BIC weight ≥ 0.95 were used for model averaging.

Relative importance of a variable, the sum of BIC weights from competing models that contained an explanatory variable, was also assessed. This metric indicated the probability that a variable was present in all top models, and/or the probability that it was included in the top set of competing models.

### Temporal cross-validation

A single year may disproportionately affect the association between WNV outcome and environmental factors. As a result, variables that are important in such a year might themselves have disproportionate weights and could thus undermine our goal of capturing uncertainty and competing hypotheses. To identify such spurious years, well known to occur in exploratory studies [54], we carried out leave-one-year-out temporal cross validation. Fifteen sets of cross-validations were conducted; for each set, data from one year between 2002 and 2016 was held out, and data from the remaining 14 years were used to rebuild the averaged model and to re-compute the variable importance. We then generated 95% confidence intervals (CI) for estimates of the variables in the new sets of averaged models and compared the results with the full model (i.e. 15-year data model).

## Results

### Seasonality of hydrology and meteorology

**Fig. 6:**
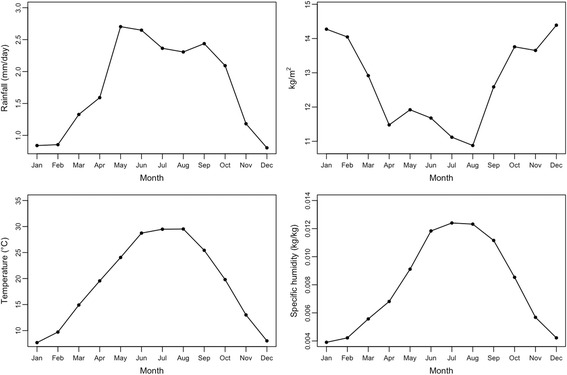
Monthly means of aggregated hydrology and meteorology in west Texas during the study period. Top left pane: rainfall; top right pane: soil moisture; bottom left pane: temperature; bottom right pane: specific humidity

West Texas hydrology and meteorology during 2002–2016 are summarized below. Monthly mean conditions followed a consistent seasonal pattern. Temperature and humidity peaked during July-August (Fig. 6). Rainfall levels peaked in May, while soil moisture conditions were maximal during December-January (Fig. 6).

### Models

A total of 12,951 models with different combinations of March to August soil moisture, rainfall, specific humidity and temperature (Table 1) were built. Of these, 3149 models had variables and intercept estimates significant at *P* ≤ 0.05. Of the 3149 significant models, 4 models, considered as the best-fitting models, were within 95% of the cumulative BIC weights and therefore used in the averaged model.

**Table 1:** Hydrology and meteorology variables of study

Variables	Unit
Average temperature per month per county	°C
Average precipitation per month per county	mm/day
Average layer 1 soil moisture per month per county	kg/m^2^
Average specific humidity per month per county	kg/kg

The best-fitting model (Table 2) showed a high relative model weight (w_i_ = 0.9014) and included March soil moisture, June temperature, August soil moisture and August specific humidity. As expected, wet conditions during spring (March soil moisture ß estimate ± standard error, SE = 0.08 ± 0.01) was a significant predictor of increased annual WNV. Also, low monthly average temperature, humidity and soil moisture conditions during late spring and summer were significant predictors of increased WNV risk, (June temperature ß estimate ± SE = -0.34 ± 0.03, August soil moisture ß estimate ± SE = -0.10 ± 0.02, August specific humidity ß estimate ± SE = -304.14 ± 42.54).

**Table 2:** Summary of the 4 best-fitting models making up the averaged model, i.e. within 0.95 of cumulative BIC weight. Total number of significant models = 3149

Model ID	Variable	Coefficient estimate	Standard error	*P*-value	BIC	Weight
1005	Intercept	94.81	9.75	2.49E-22		
	March soil moisture	0.08	0.01	4.91E-11		
	June temperature	-0.34	0.03	3.77E-25	0.00	0.9014
	August soil moisture	-0.10	0.02	9.82E-07		
	August specific humidity	-304.14	42.54	8.67E-13		
1009	Intercept	78.13	9.46	1.51E-16		
	March soil moisture	0.05	0.01	3.00E-05		
	June temperature	-0.28	0.03	5.71E-19	5.91	0.0470
	June specific humidity	187.16	44.36	2.45E-05		
	August specific humidity	-560.86	57.95	3.74E-22		
1543	Intercept	59.09	10.75	3.87E-08		
	June soil moisture	0.15	0.01	9.21E-26		
	August temperature	-0.22	0.04	9.16E-10	6.62	0.0328
	August soil moisture	-0.14	0.02	1.04E-09		
	August specific humidity	-265.29	43.96	1.60E-09		
1004	Intercept	98.58	9.75	5.16E-24		
	March soil moisture	0.09	0.01	1.67E-11		
	June temperature	-0.35	0.03	4.33E-27	7.74	0.0188
	July specific humidity	-255.70	39.40	8.57E-11		
	August soil moisture	-0.13	0.02	5.40E-12		

*Abbreviation*: *BIC* Bayesian information criterion

Three other models along with the top model accounted for 0.95 of the overall cumulative BIC weight (Table 2) and made up the top model set. The null model (i.e. containing intercept and offset term only) had a low chance of being among the competing models (*w*_*i*_ = 1.27e^-43^). Among the environmental conditions investigated, soil moisture, temperature and specific humidity were the variables in the competing models. Precipitation had a relatively low importance or low probability of appearing in the top-competing models due to high BIC values of models containing precipitation. The highest relative importance observed for a precipitation variable was 0.017 (June precipitation), but only when all 3149 significant models, instead of the top 4 models, were used to infer an averaged model (Table 3). During this rerun, changes to the relative importance of the competing model variables were negligible. Compared to soil moisture, rainfall appears to be a relatively less important variable for estimating annual human WNV cases.

**Table 3:** Relative importance of variables from all significant models (i.e. 3149). Soil moisture in March, temperature in June, specific humidity and soil moisture in August maintain their high relative importance. Precipitation showed low relative importance

Month	Variable	Relative importance	Model frequency
March	Precipitation	0.001	290
	Soil moisture	0.942	592
	Specific humidity	0.001	396
	Temperature	0.000	539
April	Precipitation	0.000	117
	Soil moisture	0.000	415
	Specific humidity	0.000	403
	Temperature	0.002	476
May	Precipitation	0.000	311
	Soil moisture	0.000	403
	Specific humidity	0.001	536
	Temperature	0.000	438
June	Precipitation	0.017	569
	Soil moisture	0.062	566
	Specific humidity	0.046	513
	Temperature	0.956	449
July	Precipitation	0.000	358
	Soil moisture	0.000	522
	Specific humidity	0.026	719
	Temperature	0.005	570
August	Precipitation	0.007	618
	Soil moisture	0.925	708
	Specific humidity	0.971	615
	Temperature	0.037	495

In the averaged model (Table 4, showing ß estimates and 95% CI), increasingly wet conditions in March and June, as well as humid conditions in June indicate increases in WNV (March soil moisture ß = 0.37 (95% CI: 0.24–0.50), June specific humidity ß = 0.26 (95% CI: 0.14–0.38), June soil moisture ß = 0.59 (95% CI: 0.48–0.70). Additionally, cooler and dryer than average conditions later in the year indicate increases in WNV as well (June temperature ß = -0.63 (95% CI: -0.75– -0.50), July specific humidity ß = -0.40 (95% CI: -0.52– -0.28), August specific humidity ß = -0.46 (95% CI: -0.60– -0.31), August soil moisture ß = -0.33 (95% CI: -0.46– -0.19), August temperature ß =-0.38 (95% CI: -0.50– -0.26).

**Table 4:** Multi-model inferred averaged model. The average confidence intervals of the estimates indicate an effect on annual WNV variability for all the variables of the multi-model (i.e. 95% CI does not include zero). The relative importance of a variable (i.e. the probability of a variable being among the best-fitting models) is high for soil moisture in March, temperature in June, specific humidity and soil moisture in August

Month	Variable	Coefficient estimate	95% CI	Relative importance
	Intercept	0.00	0.00–0.00	–
March	Soil moisture	0.37	0.24–0.50	0.97
June	Specific humidity	0.26	0.14–0.38	0.05
June	Soil moisture	0.59	0.48–0.70	0.03
June	Temperature	-0.63	-0.75– -0.50	0.97
July	Specific humidity	-0.40	-0.53– -0.28	0.02
August	Specific humidity	-0.46	-0.60– -0.31	0.98
August	Soil moisture	-0.33	-0.46– -0.19	0.95
August	Temperature	-0.38	-0.50– -0.26	0.03

*Abbreviation*: *CI* confidence interval

Table 4 also displays the relative importance of variables in the averaged model. The wBIC of competing models ranked the relative importance, which is defined as the probability of a variable being in a model with high BIC weight. Average March soil moisture, June temperature, August specific humidity and soil moisture conditions demonstrated relatively high variable importance (0.97, 0.97, 0.98 and 0.95, respectively). Variables with low relative importance in the averaged model included June specific humidity (0.05), June soil moisture (0.03), July specific humidity (0.02) and August temperature (0.03).

**Fig. 7:**
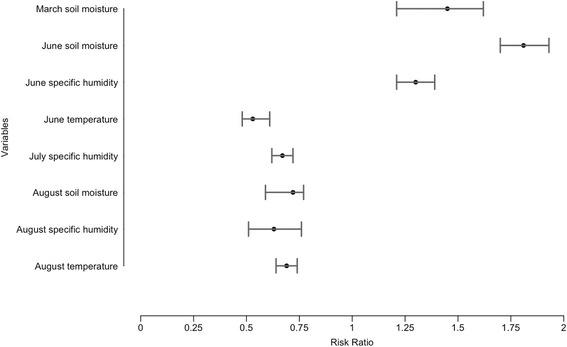
Multi-model adjusted risk ratio of WNV predictors in the averaged model, with 95% CI

Environmental conditions in the averaged model showed significant adjusted relative risks. 95% CI for both spring and summer variables were significant (Fig. 7). Spring conditions had relative risks greater than the null, indicating increases in risk of WNV transmission, whereas summer conditions showed relative risk less than 1 indicating decreases in risk of WNV transmission. For a one unit increase in March and June average soil moisture (kg/m^2^) and average specific humidity in June (kg/kg), risk of WNV transmission increases by 45%, 30% and 81%, respectively. However, for a one unit increase in June and August temperature (°C), July and August specific humidity (kg/kg), and August soil moisture (kg/m^2^), risk for WNV decreases by 47%, 31%, 33%, 37% and 28%, respectively.

Over the period of study, soil moisture had high variation (March min = 7.52 kg/m^2^, max= 18.94 kg/m^2^), (June min = 5.78 kg/m^2^, max = 17.40 kg/m^2^), (August min = 6.35 kg/m^2^, max = 14.84 kg/m^2^), with mean and standard deviation of 12.92 kg/m^2^ (± 6.09), 11.68 kg/m^2^ (± 5.14) and 10.88 kg/m^2^ (± 4.74) in March, June and August, respectively. Average specific humidity in June, July and August depicted little variation (June min = 0.009 kg/kg, max = 0.014 kg/kg; July min = 0.010 kg/kg, max = 0.014 kg/kg; August min = 0.011 kg/kg, max = 0.014 kg/kg) with mean and standard deviation of 0.012 kg/kg (± 0.002), 0.012 kg/kg (± 0.002) and 0.012 kg/kg (± 0.002), respectively. Temperature in June and August showed moderate variation (June min = 26.42 °C, max = 31.27 °C; August min = 27.55 °C, max = 32.60 °C) and spread (June temperature = 28.74 ± 2.00 °C; August temperature 29.53 ± 2.09 °C).

Putting together the maximum and minimum observed of each variable and the adjusted relative risk, these range extremes represent an increase in WNV risk of 512% for March soil moisture, 941% for June soil moisture, 0.15% for June specific humidity, 228% for June temperature, 157% for August temperature, 0.13% for July specific humidity, 0.11% for August specific humidity, and 238% for August soil moisture. Thus, given the units and ranges of the explanatory variables, soil moisture and temperature have the biggest impact on WNV risk.

**Fig. 8:**
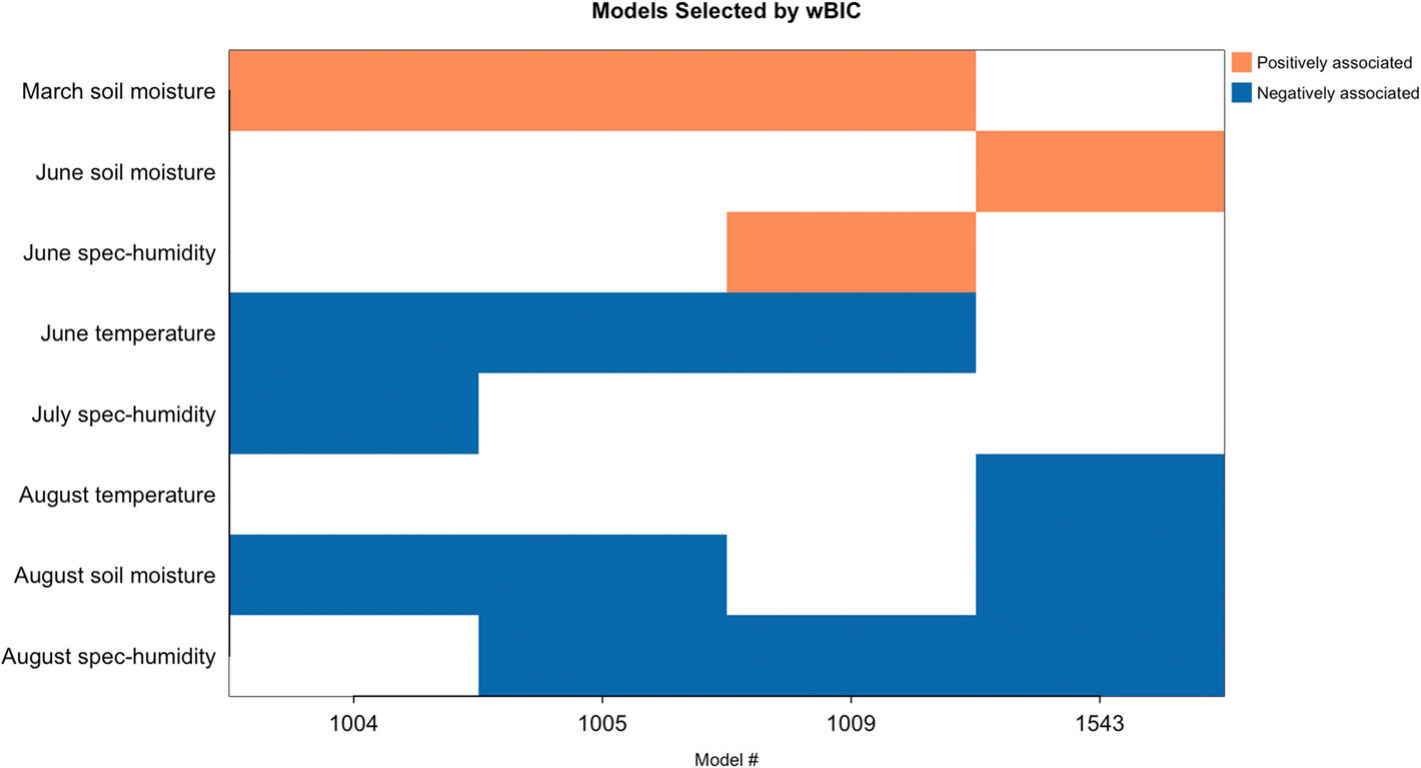
Temporal pattern of multi-model environmental conditions linked with WNV variability. In general, wetter and more humid conditions in the spring followed by drier and cooler conditions in the summer favor more human WNV cases

**Fig. 9:**
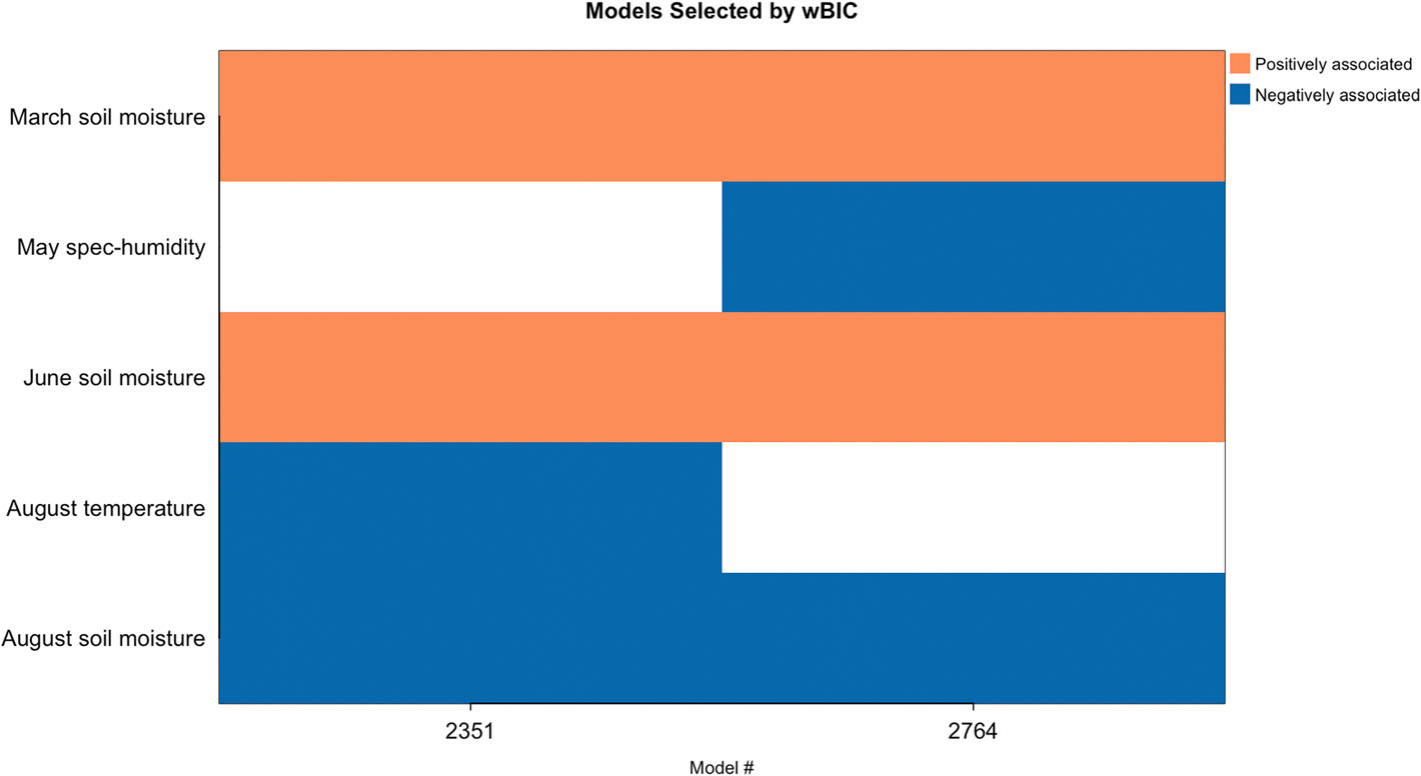
Temporal pattern of the multi-model environmental conditions linked with WNV variability (population offset omitted in models)

Overall, the multi-model inference strongly suggests that wetter and more humid conditions in spring followed by drier and cooler conditions in the summer are associated with increased annual human WNV cases (Fig. 8). Analysis of WNV variability without accounting for rate of transmission (i.e. no population offset) show generally good agreement with the above temporal pattern (Table 5, Fig. 9).

**Table 5:** Multi-model inferred averaged model of WNV, modeled without offsets term for population. There is general agreement between averaged models with and without offset terms. Wet conditions in the spring combined with dry and cool conditions are the predominating pattern in both approaches

Month	Variable	Coefficient estimate	95% CI	Relative importance
	Intercept	0.00	0.00–0.00	–
March	Soil moisture	0.45	0.33–0.58	1.00
May	Specific humidity	-0.57	-0.71– -0.43	0.38
June	Soil moisture	0.53	0.39–0.68	1.00
August	Soil moisture	-0.74	-0.96– -0.52	1.00
August	Temperature	-0.58	-0.72– -0.44	0.62

*Abbreviation*: *CI* confidence interval

### Temporal cross-validation of predictors of annual human WNV cases

**Fig. 10:**
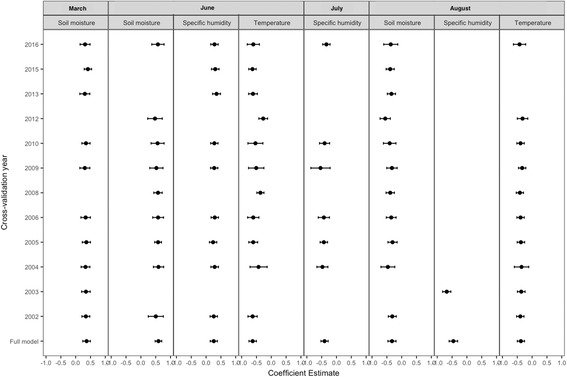
95% confidence interval of coefficient estimates of variables from cross-validation multi-models and full dataset multi-model. Specific humidity in August had the lowest probability of being included in a multi-model. Soil moisture in March and August and temperature in June and August had relatively high probability of being among the competing models. Compared to the full model estimates, the 95% CI of parameter estimates from the cross-validation sets did not vary markedly. Note: multi-models for cross-validation years 2007, 2011 and 2014 not included; only one model was within 95% of the cumulative BIC weight for each of these years

Figure 10 displays 95% CI plots, depicting changes in the coefficient estimates of variables in the averaged model during cross-validation. The confidence intervals of the variables show no marked spread or deviations. During the cross-validation analysis, a set of 15 competing models and their averaged models were generated using the cross-validation datasets. Variables of the averaged model built using the full dataset (i.e. full model), especially variables with high relative importance (March soil moisture, June temperature, August soil moisture), demonstrated high probability of being among the competing models regardless of year. Though, the probability of August specific humidity being in a top model varied greatly with year.

Years 2003 and 2012 were high outbreak periods in Texas and exclusion of data from these years allowed assessment of whether the multi-model outcomes were conditional on these years. When these years were excluded, wet soil moisture in March and June, as well as cool temperature, dry soil moisture and low specific humidity in August, remained in the set of strong predictors explaining increased WNV transmission, agreeing with the full model (Fig. 10).

A single best-fitting model (which included, March soil moisture, June temperature, August soil moisture and August temperature) had 95% of the cumulative BIC weight, for each of the following cross-validation years 2007, 2011 and 2014. As a result, a multi-model could not be inferred given the lack of competing hypotheses during the omitted years. These years might seem crucial for generating equally plausible hypotheses that are within the full data; however, use of a less conservative model selection criterion (e.g. delta BIC < 6 [55] or delta BIC < 10 [56], instead of wBIC ≤ 0.95) produced results similar to the full model (Table 6). August specific humidity showed high probability of being in the top models. Overall, several cross-validation results suggest that the observed pattern between the relatively important variables in the multi-model and human WNV risk appears robust and not solely dependent on any given year.

**Table 6:** Multi-model inferred averaged model, from dataset omitting cross-validation years 2007, 2011 and 2014. Top models selected using delta BIC < 6. Effect size, directionality, 95% CI  and relative importance of predictors closely resemble those of the predictors generated using the full dataset and 95% cumulative sum of BIC weights (Table 4)

Month	Variable	Coefficient estimate	95% CI	Relative importance
	Intercept	0	0.00–0.00	–
March	Soil moisture	0.48	0.36–0.60	1.00
June	Temperature	-0.57	-0.70– -0.44	1.00
July	Specific humidity	-0.49	-0.61– -0.36	0.78
August	Specific humidity	-0.60	-0.79– -0.40	1.00
August	Soil moisture	-0.33	-0.46– -0.19	0.95

*Abbreviation*: *CI* confidence interval

## Discussion

Multi-model inference indicates that wetter and more humid conditions in the spring in conjunction with drier and cooler summer conditions are strongly associated with increased annual cases of WNV. The use of wBIC weighted averages of best-fitting models allowed for the identification of monthly conditions strongly associated with WNV annual variability in west Texas. Cross-validation of the multi-model revealed that soil moisture in March, June and August, specific humidity in June, and temperature in June and August consistently explained human WNV case variability in west Texas more than other variables. However, soil moisture and temperature in the spring and summer appear to have the biggest impact on WNV risk.

It is worth noting that between the two variables (precipitation and soil moisture) indicating land surface water in this study, only soil moisture appeared in the best-fitting models. Soil moisture also out-performed precipitation during the cross-validation analyses, by having a higher probability of being included in a multi-model and being among the best-fitting models. These findings are likely a reflection of the reliability of soil moisture in capturing surface water availability and persistence. Rainfall can influence ponding and pooling, but unlike the near surface soil moisture employed here, it lacks feedbacks from temperature, humidity, surface pressure, soil type, vegetation type, solar and long wave radiation levels, and wind speeds [28, 29]. By accounting for these underlying effects that act on surface water availability (through the Mosaic model framework), soil moisture is more likely to provide a more robust estimate of mosquito habitat availability at the land surface than rainfall.

Our results suggest that wetter spring conditions combined with drier and cooler summer conditions strongly predict elevated human WNV infection risk. A number of studies have found similar climatic patterns describing human and mosquito WNV transmission [32, 57–59]. A study on human WNV prevalence in Colorado indicated that in the semi-arid region of Colorado, wet spring and dry summer predict human WNV; however, in the wetter regions of the state, where water-availability is high, a weak and opposite association was identified [32]. Another study found that wet spring and dry summer conditions were linked to WNV variability across the USA. However, in the semiarid environments of the southwestern USA, while a wet pattern in winter and spring were positively associated with WNV rates, temperature conditions were not significantly linked to WNV [58]. One explanation could be that given the large study area, climatic conditions varied considerably within the region. High vegetation and evapotranspiration, which are indicators of surface water availability, like soil moisture, have also been found to be positively linked to WNV fluctuations [59].

Wet and humid conditions in the spring not only provide oviposition sites and larval habitat, but also support early mosquito reproduction and avian host interactions [18, 19]. The timing of wet conditions in the year seems to be crucial to the WNV season. Wetting early in the year could lengthen mosquito-bird interactions and strengthen WNV circulation. Shaman et al*.* [57] found an association between summer and fall WNV prevalence in mosquitoes and wet soil moisture early in the spring. Among sampled *Culex* mosquito pools in Suffolk county, New York, WNV prevalence was strongly linked with wet conditions in the spring. Such early amplification of WNV in nature could result in higher spillover rates to humans, once competent avian hosts disperse in late summer [60–63].

Our results also indicate that drier and cooler than normal summer patterns strongly predict an increase in WNV transmission. During dry spells, such as droughts, birds and other wildlife tend to gather at surviving water pools. *Culex tarsalis* and *Culex quinquefacitus* are the dominant vectors of WNV in west Texas. Like most moderate-flyer mosquitoes that occupy woodlands, fields and floodlands [21], *Cx. tarsalis* may follow their hosts to converge at drying water resources [64]. At these remnant water sites, both *Cx. tarsalis* and *Cx. quinquefascitus* are capable of sustaining and dispersing WNV, because of their opportunistic feeding habits [61, 65]. Drought may also reduce predator and vector biomass [24, 66, 67], the result of which is increased survival of mosquito egg, larvae and pupae. With an already high mosquito population due to the wetter-than-normal spring, mosquito density could be high at these pools and thus raise the mosquito-bird contact rate. Several studies have shown that the congregation of avian hosts and vector mosquitoes, as occurs when water resources are limited, are linked with local zoonotic transmission and amplification of arboviruses [22, 68–70].

*Culex tarsalis,* the predominant vector of WNV in west Texas [71, 72], usually feeds on avian hosts [63, 73, 74]. However, during summer some WNV avian hosts, like ardeids [63] and passerines [61], disperse or migrate in search of food, reducing their proportion in the population and their availability for blood-feeding. The decline in host availability and peak in mosquito abundance during this time strongly favor increases in mammal and human blood-feeding [60–63]. To migrate or disperse, birds take cues from their internal body conditions, which weather and food availability influence [75–79]. In response to the growing spring vegetation, migratory or dispersing birds seeking to nest may arrive in early spring and depart in late summer in search of wintering grounds, a timing that has been proposed to be important for WNV spillover to humans [80, 81]. Cooler and drier than normal summer conditions might indicate declines in food resources and as a result reduce species abundance [70, 82–85]. A reduction in food resources may favor bird hosts aggregation at the limited food sites, reinforcing drought-induced WNV amplification.

The range of minimum temperature during the summer is typically within the limits for extrinsic incubation of WNV [12] and for mosquito development [16]. Consequently, the negative link seen in summer temperature seems to suggest a mechanism of effect on WNV transmission that is independent of virus and mosquito development. We speculate that avoidance of excessively warm temperatures by mosquitoes may contribute to these effects. Mosquito activity during cooler ambient conditions, such as at sunrise or sunset tends to be higher compared to hotter mid-day time periods [86]. Human behavior to counteract hot and humid summer days could also play a minor role in raising the risk of mosquito contact. In the summer, agricultural or recreational activities may be carried out during cooler days, or in early mornings and evenings to avoid high temperatures, during which time individuals are more likely to be exposed to mosquito biting [71], if not wearing protective gear. Therefore, cooler summer temperatures might encourage human outdoor activity but lead to increased human-vector contact rates.

Certain limitations in our study are noteworthy. Although modeled hydrology generally captures WNV activity, Mosaic hydrology model soil moisture outputs have only been validated on a regional scale. Validation of the Mosaic model was performed for sites in the southern Great Plain, which borders northwest Texas, but did not include counties from Texas. This lack of local validation introduces uncertainty from the modeled hydrology estimates. We also restricted our analysis to west Texas based on precipitation differences at the regional scale. However, ecology at the local level may still vary. Also, differences at the county level such as human activities (agricultural irrigation and occupation), not accounted for in our approach, may enhance mosquito population density [87] and mosquito-human contact rates [88]. Therefore, more work is needed to further assess the role of important WNV county-specific covariates on the observed link between hydrology, meteorology and WNV transmission risk.

## Conclusions

The findings observed here provide insight into the relationship between WNV cases, meteorology and hydrology in west Texas. The results support the idea that wetter than normal spring followed by drier and cooler than normal summer is associated with increased WNV infection risk, corroborating several studies. More so, our investigation has shown the above temporal pattern to be consistent in high and low outbreak years.

## Abbreviations

BIC: Bayesian information criterion
EIP: Extrinsic incubation period
GLM: Generalized linear model
L1SM: Layer 1 soil moisture
NLDAS: North American Land and Data Assimilation System
wBIC: Weighted Bayesian information criterion
WNV: West Nile virus
